# Health equity and system resilience during crises – ensuring healthcare for refugees based on lessons from Iran’s response to the 2021 Afghan migration

**DOI:** 10.1186/s12939-025-02564-6

**Published:** 2025-07-01

**Authors:** Zahra Karimian, Asgar Aghaei Hashjin, Saverio Bellizzi, Volker Winkler

**Affiliations:** 1 Heidelberg Institute of Global Health (HIGH), Faculty of Medicine and University Hospital, Heidelberg University, Heidelberg, Germany; 2 School of Health Management and Information Sciences, Iran University of Medical Sciences (IUMS), Tehran, Iran; 3 Public Health and Migration, World Health Organization Headquarters (WHO HQ), Geneva, Switzerland

**Keywords:** Health Equity, Health System Resilience, Universal Healthcare Coverage, Public Health Services, Migrant and Refugee Health, Displaced Populations, Undocumented Individuals, Crisis and Conflict, Eastern Mediterranean Region

## Abstract

**Background:**

Sudden influxes of displaced populations can strain health systems, especially in low- and middle-income countries. Iran hosts one of the world’s largest migrant and refugee populations –predominantly from Afghanistan – and provides inclusive access to public health and education services. In August 2021, the crisis in Afghanistan triggered a sharp increase in Afghan arrivals to Iran, raising healthcare demand amid the COVID-19 pandemic. This study examines how one of the largest public health networks in Tehran responded to this surge and the resulting impact on equitable service coverage and system resilience.

**Methods:**

We retrospectively analyzed monthly healthcare utilization data over a 13-month period (February 2021–February 2022) from the largest public health network in western Tehran, which serves approximately 5.5 million residents, including nearly 1 million migrants and refugees. Patients were categorized into six demographic subgroups: children, women of childbearing age, adolescents, young adults, middle-aged adults, and the elderly. Changes in the number and proportion of patients receiving the full Essential Health Service Package (EHSP) were assessed using six-month pre-/post-event comparisons, with statistical significance determined via chi-square tests (*p* < 0.05).

**Results:**

The total number of patients served increased eight-fold, from 88,091 in February 2021 to 717,382 in February 2022. In the six-month period following the crisis, the number of Afghan patients receiving full EHSP coverage rose by 84,522; however, the proportion of full-service coverage declined by 3.2%. Children were the most affected subgroup: despite 2,739 additional patients receiving full services, their coverage rate fell by 10.6%. In contrast, young Afghan adults experienced an increase in both number and proportion of patients receiving full services (+ 25,421, + 1.2%). All subgroup changes were statistically significant (*p* < 0.001).

**Conclusions:**

Although the public health network rapidly expanded service delivery, maintaining proportional coverage for full services proved challenging, particularly for vulnerable groups. Even established health systems with decades of experience in serving displaced populations may face transient coverage shortfalls amid demand surges compounded by pandemic-related strain. Strengthening public health emergency management through targeted resource allocation, surge capacity, and real-time monitoring of coverage indicators is essential to sustaining health equity and system resilience during future crises.

## Background

In an increasingly interconnected and crisis-impacted world, strengthening global and public health requires ensuring equitable access to healthcare for mobile communities and displaced populations [1]. The World Health Organization (WHO) Global Research Agenda on Health, Migration, and Displacement calls for greater investment in research and translating its findings into policies and practices that build inclusive and adaptive health systems [2]. Strengthening such systems not only improves health outcomes, but also aligns with international commitments to Universal Health Coverage (UHC) [3], ultimately fostering a healthier and more equitable world [2].

Health systems worldwide strive to build *resilience* – the capacity to absorb shocks and adapt to changing environments [4]. In Low- and Middle-Income Countries (LMICs), health system challenges are amplified by population growth, limited resources, and infrastructure constraints [5]. When faced with unexpected surges in patient numbers – such as those driven by crises or conflicts – these health systems often struggle to maintain both service delivery and quality for local populations and displaced groups [6]. Retrospective evaluations of system performance during past critical events can help provide insights regarding system strengths and shortcomings, guiding policymakers in better planning and developing more effective response strategies for the future [7].

Iran’s healthcare system is overseen at the national level by the Ministry of Health and Medical Education (MOHME) and is structured into a hierarchical (multi-layered) network that provides comprehensive services across the country [8]. At the provincial level, medical universities coordinate care through a network of affiliated hospitals, pharmacies, labs, and public health centers, posts and houses – all of which collaborate to streamline service delivery for residents in their respective districts / jurisdictions [9]. The system follows a three-tiered framework: primary care is provided at rural health houses and urban health posts, where community health workers provide basic preventive and curative services; secondary care is offered through district health centers and mid-sized hospitals with general and more specialized treatments; and tertiary care is delivered by university hospitals managing complex and sub-specialized medical services. All three tiers operate through referral networks that allow a smooth transition among levels to ensure equitable healthcare for all [10]. By offering specialized services in teaching hospitals and subsidizing care for vulnerable populations, the government supports a wide range of affordable health services, ranging from primary healthcare to advanced treatments [11].

Since the early 1980s, Iran has adopted a more comprehensive primary healthcare approach, emphasizing universal access, equity, and community participation [12, 13]. This initiative led to the establishment of an extensive network of rural and urban health centers, providing essential health services nationwide, which has in turn resulted in significant advancements in healthcare delivery and overall population well-being [14, 15]. These public health centers provide services to diverse demographic subgroups, prioritizing vulnerable populations – *including migrants, refugees, asylum seekers, and undocumented individuals (UIs)* – regardless of national origin, residency status and insurance coverage [16]. This is particularly noteworthy considering that according to 2024 UNHCR statistics, Iran hosts one of the world’s largest migrant and refugee populations, totaling approximately 3.8 million *documented individuals* – roughly 99% of whom are Afghans [17]. At the same time, some unofficial estimates suggest the total Afghan population in Iran may reach up to 8 million, indicating a potentially greater burden on health services than official figures reflect [18].

Despite Iran’s primary healthcare program’s achievements [19], the healthcare system has faced increasing pressure due to workforce shortages, driven in part by the increasing emigration of health professionals – an issue that was exacerbated by the COVID-19 pandemic [20] and further compounded by decades-long economic sanctions limiting resource availability [21, 22]. Therefore, ensuring both health equity and system resilience under these constraints requires a clear understanding of how the system responds when patient numbers suddenly rise, such as during large-scale migrations from neighboring countries [23].

Over the past five decades, two major events have triggered substantial cross-border migration from Afghanistan into Iran: the Soviet withdrawal in February 1989 and the U.S. withdrawal in August 2021 [24]. This study examines the consequent impact from the increase in Afghan patients from the 2021 upheaval [25] on health service coverage through one of Iran’s largest public health networks serving migrants and refugees in Tehran [26]. Therefore, the overall objective is to determine whether this sudden influx resulted in differential changes in healthcare access across various patient subgroups among Afghan migrants and refugees, and to identify which ones are most impacted and vulnerable during crises to devise appropriate strategies for their protection in the future.

## Methods

### Study setting and population

We retrospectively analyzed data from one of the three largest public health networks in the province of Tehran, affiliated with Iran University of Medical Sciences (IUMS). This network includes seven major public health centers in Western Tehran, serving approximately 5.5 million residents in the city center from districts 2, 5, 6, 9, 18, 21, and 22 [27]. In addition, the wider network provides services to residents in the suburbs of larger Tehran, as well as individuals who commute for work and school from other districts and provinces to these areas. Our patient population comprised all Afghan individuals, including migrants, refugees, asylum seekers and UIs (approximately 1 million in this jurisdiction). The population was categorized into six demographic subgroups based on age and sex: children (0–6 years), women of childbearing age (15–49), adolescents (7–17 years), young adults (18–29 years), middle-aged adults (30–59 years), and the elderly (60 + years).

### Database and Timeframe

Records of healthcare services / utilization were extracted from the public health networks’ electronic database for the 12-month window around the 15 August 2021 critical event (February 2021 through February 2022). The original dataset was recorded using the Persian calendar, which differs from the Gregorian calendar by approximately one week per month. For clarity, we approximated the timeframe to align with Gregorian months while preserving the overall 13-month observation period. The database contains a list of public health services divided into Essential Health Service Packages (EHSPs) designed specifically for each patient subgroup (tailored by age and sex). These EHSPs are developed and adapted based on recommendations from the WHO’s Package of Essential Non-communicable (PEN) Disease Interventions for primary healthcare [28, 29], as well as communicable diseases, ensuring the provision of comprehensive / holistic and patient-centered healthcare, including a range of preventive measures, screening services, and treatment interventions [30]. A concise adaptation of these EHSP categories was developed and translated from the original Persian guidelines and the electronic medical software used in public health centers. Patients who received all services from their subgroup’s respective EHSP were considered as those who received “full services” and those who received at least one service (one or more, but not all) from the list were considered “partial services.”

### Data analysis

We first generated a monthly overview of service utilization for the entire Afghan population – comprising all six demographic subgroups – to visualize trends and the magnitude of change over the 13-month period (February 2021 to February 2022). For each month, we summed the number of patients who received any EHSP service (either full or partial) across all subgroups and presented these as stacked bar charts, with full and partial service recipients distinguished. The percentage of patients receiving full services was labeled above each corresponding bar. We then produced a similar set of monthly stacked bar charts for each of the six demographic subgroups. Next, to assess changes in full-service coverage over time, we compared the percentage of patients receiving full services between the first and last months of the study period (February 2021 and February 2022), as well as immediately before and after the critical event (July 2021 vs. September 2021) – for both the overall population and each of the six subgroups. For policymaking purposes – where monthly fluctuations may be less informative for evaluating health system resilience – we also assessed the impact of the August 2021 critical event over a longer time frame by aggregating the data into two six-month periods: pre-event (February 2021–July 2021) and post-event (September 2021–February 2022). We then compared the number and proportion of fully served patients across subgroups between these periods. Chi-square tests were used to determine whether the changes in full-service coverage were statistically significant (with significance defined as p < 0.05). All analyses were conducted using R software (version 4.4.1).

## Results

Table 1 summarizes the EHSP categories for each demographic subgroup [31]. The list *mainly* includes primary healthcare services routinely provided at public health centers. Beyond standard management of non-communicable diseases (NCDs) – which may require additional tests, laboratory work, procedures, or specialist referrals – the general practitioner can also initiate screening and treatment for communicable diseases whenever clinical signs warrant further investigation. For example, a middle-aged adult patient with diabetes who visits a public health center for an insulin prescription may have their Hemoglobin A1C checked every three months, but would only undergo rapid sputum testing or chest imaging if a persistent cough raises clinical concern for tuberculosis.

**Table 1:** Overview of Essential Health Service Packages (EHSPs) by Population Subgroup

**Children (0–6)**
1. Newborn Screening (e.g., congenital hypothyroidism, phenylketonuria, etc.)
2. Childhood vaccinations
3. Evaluation and care for age- and sex- appropriate physical growth (e.g., height and weight)
4. Evaluation and care for age- and sex- appropriate mental development (e.g., speech, social skills, etc.)
5. Hearing and vision screening
6. Provision of appropriate dietary supplements (e.g., iron, vitamins, etc.)
7. Referral to a pediatric specialist, as necessary
**Women of childbearing age (15–49)**
1. Preconception care
2. Antenatal care (at least one visit per trimester)
3. Postnatal care (following live birth or Cesarean section)
4. Appropriate immunization for pregnant women
5. Referral to an obstetrician-gynecologist, as necessary
**Adolescents (7–17) – Primarily in Schools**
1. Routine immunizations for adolescents
2. Self-care education (e.g., healthy lifestyle, prevention of self-harm/suicide, etc.)
3. Oral and dental care (ages 7–14)
4. Examination (screening) for hair lice
5. Mental health screening and counseling services (e.g., by social worker)
6. Diagnosis of psychiatric disorder
7. Care by a General Practitioner (GP)
8. Referral to specialist services, as necessary
**Young adults (19–29)**
1. Routine adult immunizations
2. Screening and education for overweight and obese patients
3. Family planning and couple counseling
4. Mental health screening, diagnosis of psychiatric disorders and counseling services
5. Substance abuse and rehabilitation services
6. Care by a General Practitioner (GP)
7. Referral for specialist services, as necessary
**Middle-aged adults (30–59)**
1. Cardiovascular risk assessment (screening)
2. Diagnosis and management of diabetes
3. Diagnosis and management of dyslipidemia
4. Diagnosis and management of hypertension
5. Mental health screening, diagnosis of psychiatric disorders and counseling services
6. Care by a General Practitioner (GP)
7. Referral for specialist services, as necessary
**Elderly (60 +)**
1. Ongoing management for chronic disorders (e.g., diabetes, hypertension, hyperlipidemia, etc.)
2. Appropriate immunization services
3. Hearing and vision screening
4. Provision of dietary supplements (e.g., vitamins and minerals)
5. Screening for cancers (e.g., colorectal, prostate, breast, etc.)
6. Mental health screening and counseling services
7. Diagnosis of neurological and psychiatric disorder
8. Care by a General Practitioner (GP)
9. Referral for specialist services, as necessary

**Fig. 1:**
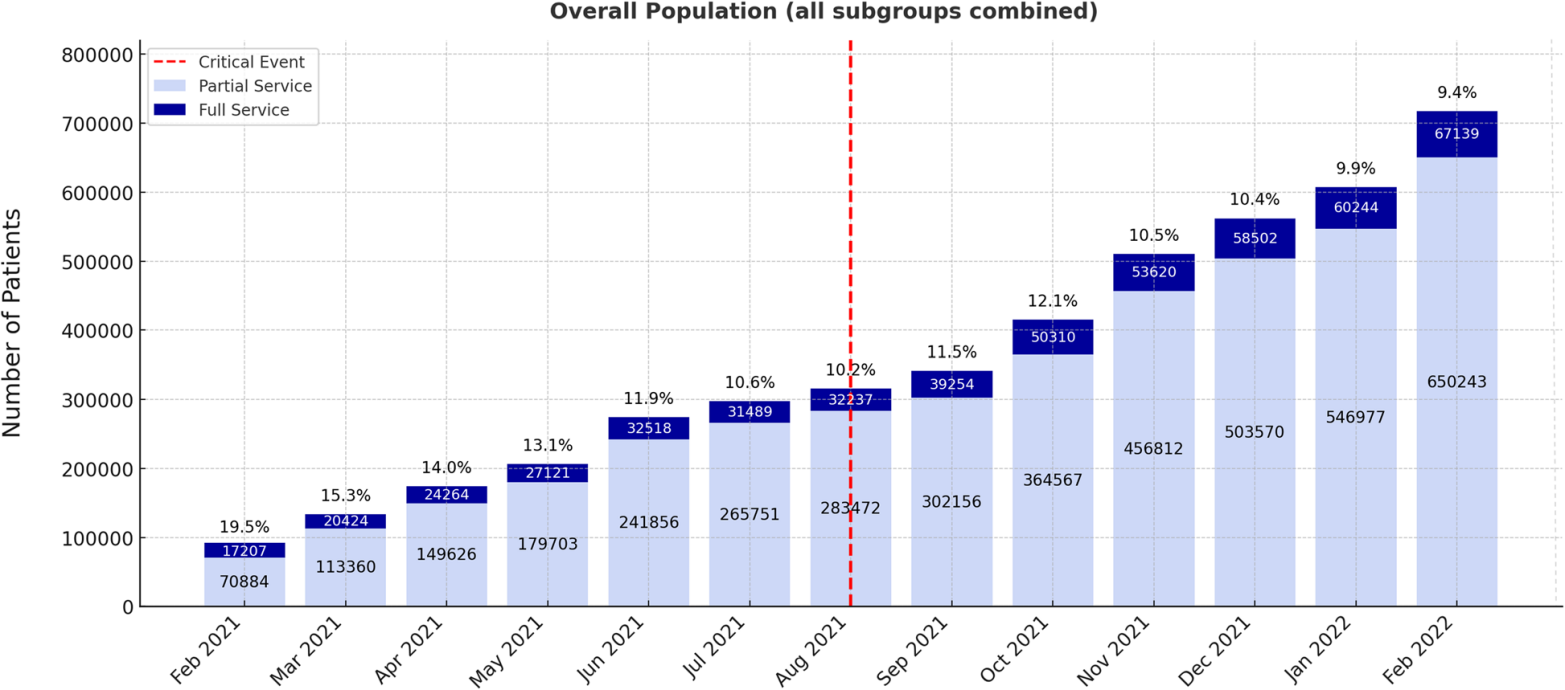
Monthly Public Health Services for Afghan Migrants and Refugees in Western Tehran – Overall Population

Figure 1 tracks monthly service use for the overall Afghan population. The total number of patients served climbed steeply from 88,091 in February 2021 to 717,382 in February 2022 (an ≈eightfold rise). Most of this growth came from partially served patients, whose numbers jumped from 70,884 to 650,243 (≈9 ×), whereas fully served patients increased from 17,207 to 67,139 (≈4 ×). The proportion of patients receiving full services (indicated above each bar) declined by more than half over the course of the year, dropping from 19.5% in February 2021 to 9.4% in February 2022 (–10.1%). The sharpest proportional drop centered on the August 2021 critical event, slipping from 10.6% in July to 10.2% in August, with only a brief recovery to 11.5% in September before continuing to decline again. Overall, the figure illustrates that although the network substantially expanded service delivery, demand grew even faster, widening the gap between partial and full coverage. Therefore, for the overall Afghan population, the predominant pattern was an upward shift in absolute numbers but a decrease in full coverage proportions – suggesting the rapid rise in overall patient demand outpaced the capacity for comprehensive service delivery.

**Fig. 2:**
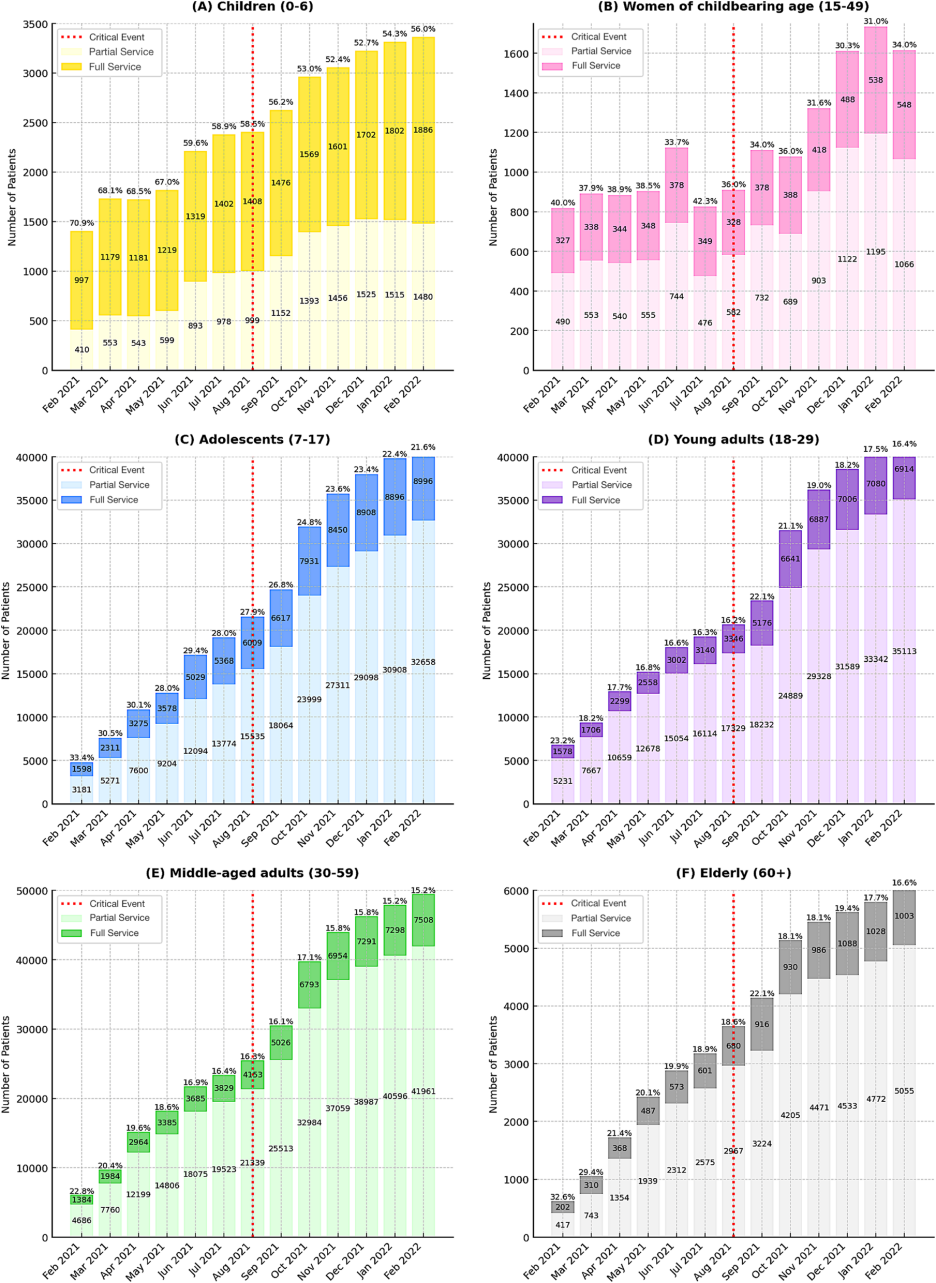
Monthly Public Health Services for Afghan Migrants and Refugees in Western Tehran – By Demographic Subgroup

Figure 2 disaggregates the monthly patterns shown for the overall population into the six demographic subgroups, revealing several contrasts that are masked in the aggregate picture. In terms of absolute patient counts, every subgroup shows a clear increase after February 2021, with the sharpest rise seen among young adults and middle-aged adults, whereas the increases are not as noticeable for women of child-bearing age and the elderly. Between February 2021 and February 2022, the proportion of children receiving full services declined from 70.9% to 56.0% (− 14.9%). Women of childbearing age showed a similar pattern, with full-service coverage falling from 40.0% to 33.9% (− 6.1%). The sharpest monthly decline in this subgroup occurred between July and September 2021, dropping from 42.3% to 34.1% (− 8.2%). Among adolescents, full-service coverage fell from 33.4% to 21.6% (− 11.8%), indicating a notable decline, although less steep than among younger children. By contrast, young adults demonstrated a relatively stable pattern, with a temporary increase from 16.3% in July 2021 to 22.1% in September 2021 (+ 5.8%), before ending the study period at 16.5% in February 2022 – below the February 2021 baseline of 23.2% (− 6.7%). Middle-aged adults saw a moderate drop in full-service coverage, from 22.8% to 15.2% (− 7.6%), suggesting that while demand rose, the ability to deliver comprehensive care lagged, particularly for working-age adults. Meanwhile, the elderly experienced the steepest decline overall, from 32.6% to 16.6% (− 16.0%), effectively halving their full coverage rate. In summary, all subgroups experienced a net decline in full-service coverage by the end of the 13-month study period.

Across all panels, the August 2021 critical event (red dashed line) marks an inflection point: patient numbers rise immediately afterwards, but the proportion of fully served either plateaus or falls – underscoring that the surge in demand stretched the capacity for comprehensive care more than for partial services. Together, the subgroup plots confirm that capacity constraints did not affect the Afghan subpopulations uniformly.

Findings from comparing the 6-month periods before-and-after the critical event are presented in Table 2. Here, we highlight the short-term effects from the critical event on the overall population followed by closer examinations of the subgroups most and least affected by the crisis. In the overall population (all subgroups combined), despite an absolute increase of 84,522 patients, the proportion of Afghan patients who received full services declined by 3.2% when comparing the period before (23.33%) and after (20.12%) the event. Children were the most affected subgroup, with their full coverage falling from 64.73% to 54.08% (–10.6), even though 2,739 more children received services post-event. Women of childbearing age exhibited a similar trend and magnitude of change as children, with an increase of 674 patients and a 5.7% decrease in full-service coverage. In contrast, young adults were the only Afghan subgroup to experience increases in both absolute patient numbers (25,421) and coverage proportion, which rose from 17.49% to 18.71% (+ 1.2%).

**Table 2:** Pre- vs. Post-Event^1^ Comparison of Comprehensive Public Health Services Provided to Afghan Migrants and Refugees in Western Tehran

Subgroups	Recipients of Full Services from EHSP^2^ (N, %)		Change in Recipients of Full Services from EHSP (N, %)
	**Feb 2021 – July 2021**	**Sep 2021 – Feb 2022**	
Children (0–6)	7297 (64.73%)	10,036 (54.08%)	+ 2,739 (-10.6%)
Women of Childbearing Age (15–49)	2084 (38.29%)	2758 (32.58%)	+ 674 (-5.7%)
Adolescents (7–17)	21,159 (29.27%)	49,798 (23.51%)	+ 28,639 (-5.8%)
Young Adults (18–29)	14,283 (17.49%)	39,704 (18.71%)	+ 25,421 (+ 1.2%)
Middle-aged Adults (30–59)	17,231 (18.28%)	40,870 (15.84%)	+ 23,639 (-2.4%)
Elderly (60 +)	2541 (21.39%)	5951 (18.48%)	+ 3410 (-2.9%)
Overall Population (Combined Subgroups)	64,595 (23.33%)	149,117 (20.12%)	+ 84,522 (-3.2%)

1. Critical Event: 15 August 2021, withdrawal of US-led foreign forces from Afghanistan and return of the Taliban to power

2. EHSP: Essential Health Service Package

^*^The *p*-values for the chi-square test results were significant (*p* < 0.001) for the before and after comparisons of all population subgroups

## Discussion

The main findings of our study are presented in the context of the critical event's impact on the population’s access to public health services and its implications for the healthcare system.

### 1. Impact on health equity: population-level and subgroup disparities

i. Overall Afghan population (all subgroups combined): As shown in Fig. 1, the absolute number of Afghan patients fully served rose in the overall population over 13 months, although their proportional coverage generally declined (+ 49,932, –10.1%). This reflects a health system that expanded in terms of absolute capacity but could not keep pace with rising demand. The six-month pre-/post- comparison (Table 2) reinforces this trend, showing a statistically significant drop in full-service coverage despite an increase in number of patients served (+ 84,522, –3.2%). Basically, despite the public health system’s continuous efforts to keep up with the surge in demand, it could not catch up with the sudden, large, and continued increase of patients over this period. Conversely, while full-service coverage declined, the proportion of patients receiving partial services increased over the same 13-month period (+ 579,359, + 10.1%). This shift may be partly attributed to the influx of foreign nationals without legal status – such as UIs – who often lack formal documentation or insurance coverage, making it difficult for public health centers to monitor service delivery and ensure proper follow-up [32].
  We speculate that as in most crises, many Afghans who fled after the 2021 upheaval entered Iran without legal residency [33]. While the Iranian healthcare system does not deny UIs access, it cannot consistently monitor or accurately document whether unregistered individuals receive full EHSP coverage [34]. By contrast, Afghan migrants and refugees with legal status (e.g., Amayesh cardholders) can enroll in the national Universal Public Health Insurance (UPHI), enabling more structured service delivery and better service documentation [35]. Although health access for UIs remains limited relative to insured migrants and refugees, Iran’s model is comparatively inclusive, since evidence indicates that in many host countries UIs are generally not even eligible for any health services [32, 36]. In fact, findings from UNHCR’s 2021 health-inclusion survey including 49 countries showed that only 77% include refugees in their national health plans, and even then, the scope of services varied considerably [37]. Iran is therefore notable for at least extending public health insurance coverage to documented migrants and refugees under a scheme similar to that offered to its own citizens, even though significant gaps remain for UIs [38].
ii. Trends and magnitude of change across subgroups: As shown in Fig. 2 and Table 2, young adults were the only Afghan subgroup to exhibit a consistent upward trend in the number of patients served over the 13-month study period, along with increases in both absolute numbers and the proportion fully served in the six-month pre-/post-event comparison (+ 25,421, + 1.2%). This finding aligns with literature suggesting that younger adults can more readily access host services, possibly due to fewer barriers (e.g., greater mobility, higher literacy rates, and a faster adaptation to language and cultural norms) compared to other demographics [39–41]. Therefore, the slight improvements seen among the least-impacted (most protected) subgroup could be mostly attributed to differences in healthcare-seeking behaviors.
  Children (0–6 years) experienced the largest drop in full coverage proportion in the six months following the event (–10.6%), highlighting the particular vulnerability of pediatric groups in crisis settings [42, 43]. This warrants attention since research shows that in emergencies and humanitarian contexts, children of all age groups – especially those under 5 – are the most affected part of the community, often experiencing mortality rates 2–70 times higher than average [44].
  Moreover, women of childbearing age also experienced a notable decline in full coverage in the post-event period (–5.7%), suggesting a shared vulnerability with children. Since Maternal and Child Health (MCH) are so closely intertwined / interdependent, we should pay closer attention to Afghan women of childbearing age, who often give birth at home without trained attendants and consequently face higher risks of medical complications [45]. One might have expected higher rates of full-service coverage among Afghan women, given the enactment and implementation of national pronatalist policies mandating improved clinical support and medical services for both antenatal and postnatal care [46, 47]. Yet, the findings suggest that a parallel protective mechanism is still needed for refugee and undocumented women of childbearing age to prevent disproportionate service gaps compared to the host population – acknowledging this subgroup’s dual vulnerability [41]. This observation underscores the need for dedicated funding streams and government partnerships with donors, including NGOs and international agencies such as UNFPA and UNICEF to ensure equitable maternal health coverage for displaced populations [48, 49].
iii. Statistical versus practical significance: As noted in Table 2, the p-values for pre- and post-event comparisons of all subgroups, as well as the overall population, were highly significant (p < 0.001), which may reflect large sample sizes and/or meaningful shifts [50]. Regardless, some of the observed changes – such as the 10.6% drop in full coverage among Afghan children – appear substantial enough to indicate practical significance, as they represent a large number of individuals who did not receive full health coverage. Specifically, 1,063 fewer children received full coverage in the six months following the critical event.

### 2. Implications for health system resilience: barriers, strain and adaptation

i. Access barriers and social determinants: The Iranian healthcare system provides free-of-charge treatment for communicable diseases (such as tuberculosis, hepatitis C, and HIV) to all individuals, including those who are uninsured or undocumented [51]. However, as observed in other studies on displaced populations, controlling communicable diseases among newly arrived migrants and refugees is hampered by vaccine hesitancy [52] and poor adherence to antimicrobial treatments [53]. Vaccine hesitancy can lead to outbreaks even among previously vaccinated individuals [54], while incomplete treatment can drive antimicrobial resistance in the broader population [55]. These concerns are especially pronounced among UIs, who often avoid visiting public health centers for fear of being reported to immigration authorities (deported), do not complete immunization series for their children and lack sufficient education / awareness of the serious consequences of untreated infections – resulting in poor adherence to prescribed treatments [56]. Many also lack stable housing or reliable contact information, making follow-up difficult and causing potential inefficiencies such as duplicate services or missed care [57]. This issue is especially concerning since UNHCR estimates that roughly one-third of Afghans in Iran are undocumented, with 40% of them under the age of 18 [58, 59]. Addressing these barriers through tailored educational programs, targeted outreach efforts, and more inclusive insurance options will be essential for the Iranian health system to protect the health of *both* migrant and host communities [60, 61].
ii. System strain and spillover to the host population: The Eastern Mediterranean Region (EMR) has the highest number of refugees and internally displaced persons (IDPs) among all WHO regions. In 2021 alone, more than half of the world’s refugees (27.1 million) originated from the EMR, primarily from Syria and Afghanistan [62]. Other studies which have examined the effects of large-scale migrant and refugee influxes from the Region (e.g., Palestinian and Syrian refugees in Lebanon and Jordan) have reported that acute surges of new arrivals have led to resource constraints for both incoming and host populations [63–65]. More specifically, host countries often experience a substantial rise in demand for healthcare services during large influxes of migrants, which can lead to overcrowded facilities, extended wait times, and difficulties in providing adequate and / or quality care [66]. Although our study focused on Afghan nationals alone, the marked increase in patient numbers during the post-event period suggests considerable strain on the Iranian public health system. Furthermore, while our study does not include direct comparisons with the host community, the noticeable decline in full-service coverage – visible in both the monthly trends (Figs. 1 and 2) and in the six-month pre/post analysis (Table 2) – point to broader system-level pressures. Such strain warrants careful attention from health officials, since in an overstretched health system service access likely becomes limited for migrants, refugees, and citizens alike.
iii. Long-term monitoring and adaptive capacity: We analyzed only a 13-month window surrounding the August 2021 crisis to capture the immediate, short-term impact on the public-health network. While the steady monthly rise in the number of patients served suggests that the system initially expanded capacity in response to the surge, global evidence and guidance emphasize that longer-term monitoring of the health system is required to determine health system resilience, particularly in the context of protracted crises such as large-scale migration and ongoing refugee inflows [67–69]. A review of Jordan’s response to the Syrian refugee crisis illustrates key parallels with Iran [70]. It notes that displacement commonly lasts decades and that about 78% of refugees worldwide live in host communities (outside of recognized refugee camps) – relying on the same health services as the local population [70, 71]. Furthermore, similar to most Afghans in Iran, the study notes that large-scale refugee returns to their home country are often unlikely in the foreseeable future, underscoring the need to strengthen host-country health systems once immediate humanitarian needs are met [70, 72]. Additionally, in prolonged displacement settings – such as the Rohingya settlements in Bangladesh – initial service surges may strain the system, but they are often followed by gradual adaptation [73, 74]. These examples all illustrate that short-term observations in our six-month pre/post comparison may not predict long-term performance of the health system and that sustained monitoring will be essential to determine whether the public health network ultimately recovers, stabilizes, or develops widening gaps that persist in care delivery.

### 3. Health Programs and Policy Recommendations

i. Expand inclusive health insurance programs and financing mechanisms: Iran already covers documented migrants and refugees through its UPHI. Extending affordable insurance plans or interim voucher schemes to UIs could lower administrative barriers, improve follow-up, and reduce inefficiencies (such as duplicate or missed services) which could in turn help relieve pressure on an already strained healthcare system [16, 34].
ii. Prioritize surge capacity for vulnerable groups: The steep decline in full-service coverage among children and other high-risk, vulnerable groups (e.g., women and the elderly)– despite the rise in the number of patients served – underscores the need for real-time monitoring and rapid surge response. Digital tools that provide subgroup-specific utilization data can alert managers to early declines, enabling prompt interventions and more strategic resource allocation during health emergencies [75, 76]. When dashboards identify a drop in coverage, policymakers should activate pre-planned surge measures such as targeted staff redeployment, expanded immunization programs, pediatric outreach, and mobile clinics [77, 78].
iii. Establish standardized, inclusive health information systems: Health information remains fragmented in many EMR Member States, including Iran, due to lack of a standardized and comprehensive platform that captures demographic details for refugees, migrants, and UIs, as well as citizens [79]. An integrated, centralized national data system aligned with the Global Research Agenda on Health could ensure more efficient use of limited resources and support evidence-informed policymaking – especially during crises – in a region where 73% (16 of 22) of Member States are LMICs [80].

### Strengths and limitations

This study is notable for its use of real-world data from one of the largest public health networks serving Afghan migrants and refugees in the capital, providing a comprehensive perspective on how a crisis can influence multiple patient subgroups while the health system is under significant pressure. Therefore, it offers insights relevant not only for Iranian policymakers but also for other LMICs facing similar constraints.

Despite its strengths, a few limitations warrant consideration. First, COVID-19 likely had a significant impact on service delivery and utilization at public health centers during this period through lockdown policies, changes in health-seeking behavior, staff shortages, and a staggered vaccine rollout that differed by patient subgroup [81]. Moreover, since COVID-19 immunization services were accessible to *both* host and displaced populations, some of the post-event increases in patient numbers (particularly in the later phase of our study period) may reflect a vaccine-driven rebound as individuals resumed seeking care at public health facilities following the relaxation of social distancing policies – rather than being solely attributable to the influx of migrant patients [82, 83]. Second, even though an Interrupted Time Series (ITS) approach might have better captured monthly fluctuations, the lack of disaggregated COVID-19 vaccination data by population subgroup and health center limited this option. Thus, a simpler pre/post 6-month comparison was chosen to meet immediate policymaking needs [84–86]. Finally, underreporting due to UIs may mean actual demand and services rendered were even higher than those reported by the centers.

Future research should evaluate and track longitudinal health service data among both migrants and refugees, as well as host populations during crises; incorporate qualitative insights from patients and healthcare workers to inform balanced solutions to service delivery barriers; and use comparative analyses across provinces or neighboring countries to identify best practices for crisis response in settings with large displaced populations [87].

## Conclusions

Addressing health equity requires a global commitment to integrating migrant and refugee health into national public health strategies. Our findings show that, despite Iran’s well-established health system and decades of experience serving displaced populations, it experienced temporary coverage gaps when confronted with a sudden surge in demand following the August 2021 crisis in Afghanistan. Notably, the most vulnerable subgroups faced the greatest declines in proportional full-service coverage. This challenge is further compounded when the health system is simultaneously strained by multiple other pressures, such as pandemics, workforce shortages, and resource constraints. Strengthening public health emergency management through enhanced surge capacity, targeted resource allocation, and systematic monitoring of coverage indicators are critical to sustaining a resilient health system in the face of future crises.

## Data Availability

The datasets used in this study are part of a larger public health database that is not publicly available to safeguard patient privacy and confidentiality. However, they may be made available by the corresponding author upon reasonable request.

## Abbreviations

COVID-19: Coronavirus Disease-2019
EMR: Eastern Mediterranean Region
IDP: Internally Displaced Person
ITS: Interrupted Time Series
LMICs: Low- and Middle-Income Countries
MCH: Maternal and Child Health
MOHME: Ministry of Health and Medical Education
NCD: Non-Communicable Disease
NGO: Non-Governmental Organization
UI: Undocumented Individual
UHC: Universal Health Coverage
UNHCR: United Nations High Commissioner for Refugees
UNFPA: United Nations Population Fund Agency
UPHI: Universal Public Health Insurance
WHO: World Health Organization
